# Multidisciplinary transmural rehabilitation for older persons with a stroke: the design of a randomised controlled trial

**DOI:** 10.1186/1471-2377-12-164

**Published:** 2012-12-31

**Authors:** Tom PMM Vluggen, Jolanda CM van Haastregt, Jeanine A Verbunt, Elly JM Keijsers, Jos MGA Schols

**Affiliations:** 1 CAPHRI School for Public Health and Primary Care, Maastricht University, PO box 616, Maastricht, MD, 6200, The Netherlands; 2 Department of Health Services Research, Maastricht University, PO box 616, Maastricht, MD, 6200, The Netherlands; 3 Department of Rehabilitation Medicine, Maastricht University, PO box 616, Maastricht, MD, 6200, The Netherlands; 4 Department of General Practice, Maastricht University, PO box 616, Maastricht, MD, 6200, The Netherlands; 5 Faculty of Health Medicine and Life Sciences, CAPHRI School for Public Health and Primary Care, Department of Health Services Research, Maastricht University, PO box 616, Maastricht, MD, 6200, The Netherlands

**Keywords:** Stroke, Rehabilitation, Aftercare, Elderly persons, Discharged, Nursing home

## Abstract

**Background:**

Stroke is one of the major causes of loss of independence, decreased quality of life and mortality among elderly people. About half of the elderly stroke patients discharged after rehabilitation in a nursing home still experience serious impairments in daily functioning one year post stroke, which can lead to difficulties in picking up and managing their social life. The aim of this study is to evaluate the effectiveness and feasibility of a new multidisciplinary transmural rehabilitation programme for older stroke patients.

**Methods:**

A two group multicentre randomised controlled trial is used to evaluate the effects of the rehabilitation programme. The programme consists of three care modules: 1) neurorehabilitation treatment for elderly stroke patients; 2) empowerment training for patient and informal caregiver; and 3) stroke education for patient and informal caregiver. The total programme has a duration of between two and six months, depending on the individual problems of the patient and informal caregiver. The control group receives usual care in the nursing home and after discharge.

Patients aged 65 years and over are eligible for study participation when they are admitted to a geriatric rehabilitation unit in a nursing home due to a recent stroke and are expected to be able to return to their original home environment after discharge. Data are gathered by face-to-face interviews, self-administered questionnaires, focus groups and registration forms. Primary outcomes for patients are activity level after stroke, functional dependence, perceived quality of life and social participation. Outcomes for informal caregivers are perceived care burden, objective care burden, quality of life and perceived health. Outcome measures of the process evaluation are implementation fidelity, programme deliverance and the opinion of the stroke professionals, patients and informal caregivers about the programme. Outcome measures of the economic evaluation are the healthcare utilisation and associated costs. Data are collected at baseline, and after six and 12 months. The first results of the study will be expected in 2014.

**Trial registration:**

International Standard Randomised Controlled Trial Register Number ISRCTN62286281, The Dutch Trial Register NTR2412

## Background

Stroke is one of the major causes of loss of independence, decreased quality of life and mortality among elderly people [1,2]. Each year, about 45,000 people in the Netherlands suffer from stroke and associated functional impairments [3]. Almost 56% of stroke patients are 65 years or older [4].

In contrast to other countries, nursing homes in the Netherlands fulfil an important role in the rehabilitation of older stroke patients [5]. In the Netherlands, 31% of stroke patients are admitted to a geriatric rehabilitation unit in a nursing home after hospital discharge [6-8]. Stroke patients in general are admitted to a geriatric rehabilitation unit in a nursing home when they are over 65 years of age and have coexisting multimorbidity, which means that they are incapable of completing an intensive neurorehabilitation programme in a regular rehabilitation centre.

About half of the stroke patients discharged home after rehabilitation in a nursing home still experience serious impairments in daily functioning one year post stroke, that complicate fulfilling their former social roles [9-12]. Common residual problems of elderly stroke patients are emotional and psychological problems such as depression or cognitive deficits, social problems and health-related problems including rest paralysis and fatigue [13-17]. Besides having negative consequences for the patients, these problems may also increase the care burden and decrease the quality of life of their informal caregivers [18].

Currently, in the Netherlands there is a lack of tailor-made, specialised multidisciplinary aftercare following rehabilitation in nursing homes [19]. This may result in inadequate coping skills with the remaining physical, cognitive and/or psychosocial impairments in their home environment [20,21]. These problems may lead to difficulties in the performance of normal day-to-day activities, fulfilling former social roles, maintaining the functional level which has been achieved in the nursing home, and may have negative influence on the burden of care and quality of life of the patient and informal caregiver [22-24]. Eventually, permanent admission to a residential care facility or nursing home could become necessary. However, tailor-made multidisciplinary aftercare may prevent this and contribute to elderly stroke patients living independently in the community as long as possible.

To date, there is no effective aftercare programme available [25]. But research findings in the field of stroke aftercare suggest that adequate aftercare should include, after discharge, follow-up treatment in the patients’ home environment which improves personal independence in daily living [26]. Furthermore, it should include strategies to increase the skills to cope with the remaining physical, cognitive and/or psychosocial impairments, to improve social participation and to maintain functional level after rehabilitation [27]. Support for the informal caregiver is important to decrease the burden of care and improve quality of life.

Based on consideration of shortcomings in current stroke care for older stroke patients and the improvements as suggested in the literature, a multidisciplinary rehabilitation programme for older stroke patients is proposed. This paper presents the design of a multicentre trial evaluating a new multidisciplinary rehabilitation programme for older stroke patients admitted to a geriatric rehabilitation unit of a nursing home.

### Objectives

The primary aim of this study is to evaluate the effect of this new rehabilitation programme on the level of daily activity, functional independence, perceived quality of life and social participation in elderly stroke patients as compared with usual care. In addition, the effect of the programme on the perceived care burden and quality of life of the informal caregiver is assessed.

The aim of the process evaluation is to gain insight into implementation fidelity, programme deliverance and the opinion of the stroke professionals, patients and informal caregivers about the programme. The aim of the economic evaluation is to assess the effects of the programme on health care utilisation and associated costs of elderly stroke patients.

## Methods

### Study design

**Figure 1:**
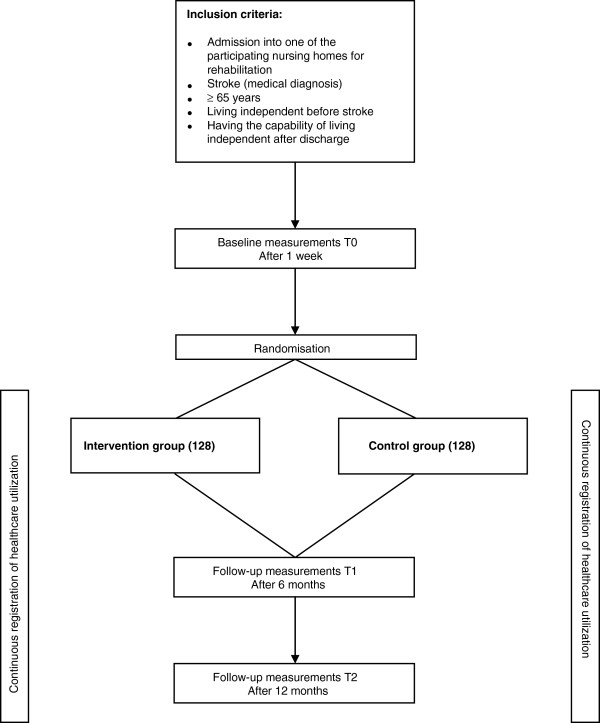
Design of the study evaluating the multidisciplinary rehabilitation programme.

The design of this study is a multicentre randomised controlled trial with patients allocated to either an intervention or control group. The study design is presented in Figure 1. The study consists of an effect, process and economic evaluation, and will be carried out in the south of the Netherlands. Eight nursing homes with a specialised geriatric rehabilitation unit for stroke patients participated in this study.

The study and research protocol have been approved by the medical ethics committee of the university hospital Maastricht and Maastricht University (MUMC+), the Netherlands.

### Study population

The study population consists of stroke patients and their primary informal caregivers. The inclusion of patients starts directly after the acute hospital phase. Patients are eligible to participate in the study when they meet the following inclusion criteria: admission to one of the participating geriatric rehabilitation units due to a recent stroke, aged 65 years or over, living independently in the community before a stroke, expected to be able to return home after discharge and giving informed consent to participate. The multidisciplinary teams of the participating units will check whether patients fulfil the inclusion criteria. The teams usually consist of a nursing home physician, a physiotherapist, an occupational therapist, a speech therapist and a psychologist. If the patient and the informal caregiver are unable to give informed consent, or the patient is medically unstable and not able to start rehabilitation, the patient will be excluded. In addition, for every participating patient his/her primary informal caregiver is invited to participate in the study. A person is considered to be the primary informal caregiver in case the patient indicates him/her as the person mostly involved in informal and social care related activities on a long term basis.

### Randomisation

The randomisation procedure is conducted by a research assistant, who is not involved in the geriatric rehabilitation care. Randomisation is performed based on a computerised block randomisation schedule (block size 8) to allocate eligible patients to the intervention or control group in each of the participating nursing homes. Participants allocated to the intervention group receive the multidisciplinary rehabilitation programme and participants allocated to the control group receive usual care.

### Blinding

The participating nursing homes, patients, informal caregivers and the multidisciplinary teams who are participating in the study are not blinded for the treatment allocation. Research assistants involved in data collection and data analyses are blinded for treatment allocation.

### Intervention

#### Description of the multidisciplinary rehabilitation programme for older stroke patients

##### Organisation

The rehabilitation programme consists of the following three care modules: 1) neurorehabilitation treatment for elderly stroke patients; 2) empowerment training for patient and informal caregiver; and 3) stroke education for patient and informal caregiver. The total programme, including all three modules, has a duration of between two and six months, depending on the individual problems of the patient and informal caregiver.

At the start of the programme an individual treatment plan is made including rehabilitation goals facilitating the transition from in- to outpatient rehabilitation care and to guide further rehabilitation at the patient’s home. The individual patient’s goals will be leading the treatment during both the in-patient and home-based rehabilitation. Rehabilitation goals during the total programme are formulated based on the Goal Attainment Scaling (GAS) method. GAS appeared to be an appropriate method as a guide for rehabilitation treatment for elderly people [28]. Both patient and informal caregiver receive a tailor-made treatment programme to improve their individual level of functioning. To evaluate the treatment progress multidisciplinary team meetings will be organised every four to six weeks in the nursing home. To facilitate optimal communication and information distribution an electronic transmural patient record will be used.

#### The transmural stroke care coordinator

In order to facilitate the continuity of care, in the proposed programme a transmural stroke care coordinator is introduced as a new rehabilitation team member. He/she facilitates the transition of nursing home rehabilitation care services to community care by supporting the collaboration between the multidisciplinary stroke team of the nursing home and the community health services, namely community nurses, paramedical professionals and the general practitioner. After discharge, the coordinator conducts home visits, supports the general practitioner by organising multidisciplinary stroke team meetings and guides the patient and informal care giver in learning to apply self-management principles.

##### Module 1: neurorehabilitation treatment for elderly stroke patients

This module will focus on (re)learning the abilities needed for individual patients to function as independently as possible in their home environment. To optimise recovery, increase independence and check whether patients’ home needs any modification before discharge, an occupational therapist and physical therapist will train the patients during guided home visits in their own home environment when they are still staying in the nursing home [29]. The care within this module is conducted by a multidisciplinary stroke team consisting of nursing home professionals, including a nursing home physician, a physiotherapist, an occupational therapist, a speech therapist, a psychologist and a transmural stroke coordinator. Besides treatment, this part of the programme includes all actions needed to ensure further aftercare, as well as activities to facilitate procedures for necessary home adaptations and assisting devices [30].

##### Module 2: empowerment training for patient and informal caregiver

This module begins after discharge to the home environment where the treatment focus will switch to learning to cope with residual impairments as a result of a stroke. Both patients and informal caregivers will be trained by the transmural stroke coordinator in improving their coping strategies and empowerment techniques based on self-management [27,31]. The care in this module will only be given by the professionals of the multidisciplinary team of the nursing home, who are involved in the treatment based on the individual needs of the patient. The transmural stroke coordinator coordinates care in collaboration with the general practitioner.

##### Module 3: stroke education for patient and informal caregiver

The last module is a stroke education course organised for patients and their informal caregivers. This course consists of four meetings with the focus on respectively the psychological and emotional consequences of stroke, perceived problems in living independently and returning to society and the new role of the healthy partner as caregiver. The module will be provided by a neuropsychologist, two volunteers of the Dutch Stroke Patient Association and Informal Caregivers Association and a social worker. The education course is organised in cooperation with the Dutch Stroke Patient Association and Informal Caregivers Association. In this part of the intervention the transmural stroke coordinator is responsible for inviting the patients and informal caregivers to the course.

##### Usual care

The usual care of elderly stroke patients after hospital discharge consists of a multidisciplinary neurorehabilitation programme in a nursing home. Most usual care programmes focus more on the needs of the patient than the informal caregiver. Usual care is provided by a multidisciplinary stroke team also containing a nursing home physician, a general practitioner, a physiotherapist, an occupational therapist, a speech therapist and a psychologist. A transmural stroke care coordinator is not involved. After discharge the follow-up care is provided separately by community services. The medical and paramedical information about the patient is distributed by letter. The main differences between the new programme and usual care are presented in Table 1.

**Table 1:** Content differences between multidisciplinary rehabilitation programme and usual care

	**Multidisciplinary transmural programme**	**Usual care**
***Care content***		
Multidisciplinary stroke team	+	+
Care based on Dutch stroke guidelines	+	+
Tailored approach with Goal Attainment Scaling	+	-
Self-management	+	-
Stroke education	+	-
Home therapy during nursing home admission	+	-
Multidisciplinary outpatient rehabilitation	+	-
Home visits of transmural stroke care coordinator	+	-
***Care organisation***		
Transmural stroke care coordinator	+	-
Multidisciplinary team meetings in nursing home	+	+
Multidisciplinary team meetings after discharge	+	-
Electronic transmural patient record	+	-

### Outcome measures

#### Effect evaluation

##### Primary outcome measures for patient

An overview of all outcome measurements per time point is presented in Table 2. Primary outcome measures are daily activity measured by means of the Frenchay Activity Index (15-items activity scale) [32], functional dependence measured by means of the Katz-15 (15-items ADL and IADL scale) [33], perceived quality of life measured by means of the Stroke Specific Quality of Life scale (49-items stroke specific quality of life scale) [34] and social participation measured by means of two subscales (autonomy outdoors and social life and relationships) of the Impact on Participation and Autonomy (scale about participation in everyday life) [35].

**Table 2:** Primary and secondary outcome variables of the effect evaluation per time point

**Outcome variables**	**Scale**	**No.of items**	**T0**	**T1**	**T2**
***Primary outcome variables* (*patient*)**					
Activity level after stroke	Frenchay Activity Index	15	FI	FI	FI
Level of functioning	Katz-15	15	FI	FI	FI
Quality of life (stroke specific)	Stroke Specific Quality of Life questionnaire	49	FI	FI	FI
Social participation	Impact on Participation and Autonomy (subscales autonomy outdoors and social life and relationships)	12	FI	FI	FI
***Secondary outcome variables* (*patient*)**					
Perceived health	Question 1 and 2 RAND-36	2	FI	FI	FI
Mental wellbeing	RAND-36 (subscale mental wellbeing)	5	FI	FI	FI
Social functioning	Question 10 RAND-36	1	FI	FI	FI
Quality of life	Question 1 and 2 RAND-36 and a mark for quality of life	3	FI	FI	FI
Process questionnaire patient	-	24/15	-	FI	FI
Process questionnaire informal caregiver	-	21/14	-	SQ	SQ
Cost questionnaire	-	34	FI	FI	FI
***Outcome variables* (*informal caregiver*)**					
Perceived care burden	Self-Rated Burden VAS and Carer QoL	10	SQ	SQ	SQ
Objective care load	Erasmus iBMG	4	SQ	SQ	SQ
Quality of life	Question 1 and 2 RAND-36 and a mark for quality of life	3	SQ	SQ	SQ
Perceived health	Question 1 and 2 RAND-36	2	SQ	SQ	SQ
***Additional outcome measures***					
Background characteristics patient	-	10/5/5	FI	FI	FI
Background characteristics informal caregiver	-	8/7/7	SQ	SQ	SQ
Cognitive functioning patient	Mini Mental State Examination	12	FI	-	-

T0 = at baseline, T1 = after 6 months, T2 = after 12 months, FI = face-to-face interview, SQ = self-report questionnaire.

##### Secondary outcome measures for patient

Secondary outcome measures are perceived health measured by question 1 and 2 of the RAND-36 (generic quality of life scale), mental wellbeing measured by subscale mental wellbeing of RAND-36, social functioning measured by question 10 of RAND-36 and quality of life measured by question 1 and 2 RAND-36 and a mark for quality of life.

##### Outcome measures related to informal caregiver

Outcome measures are the perceived care burden measured by means of the Self-Rated Burden VAS (care burden vas scale) and the Carer QoL (carer quality of life scale) [36], objective care burden measured by means of the Erasmus iBMG (4-items care burden scale) [37], quality of life and the perceived health both measured by question 1 and 2 of RAND-36 (including a mark for quality of life) [38].

##### Additional outcome measures

Besides the primary and secondary outcomes, the following background characteristics are measured in both patients and informal caregivers: age, gender, social economic status, ethnicity, level of education, marital status, living situation, travelling distance to patient and relationship with patient. In the participants cognitive functioning is also measured at baseline by means of the Mini Mental State Examination (12-items dementia scale) [39].

##### Process evaluation

In every participating nursing home a process evaluation will be conducted in order to study factors influencing the effectiveness and feasibility of the programme and to identify potential influencing factors that can facilitate future implementation of the intervention. The process evaluation will be based on the method suggested by Saunders et al. [40] with main evaluation themes: implementation fidelity, programme delivery and the opinions of the stroke professionals, patients and informal caregivers. First, implementation fidelity will be studied by evaluating the extent to which the implementation of the programme was performed as planned. Second, programme delivery is evaluated by checking whether rehabilitation care as provided by the stroke professionals has indeed been performed according to the study protocol. Third, satisfaction of the stroke professionals, patients and informal caregivers with the programme is evaluated by assessing their opinion on various programme elements as performed.

##### Economic evaluation

The evaluation of the rehabilitation programme also involves a cost-effectiveness analysis in which we compare the programme costs and additional healthcare costs with those of usual care. The care utilisation is measured by continuously recording the volumes of health care utilisation consisting of costs for hospital admissions, structural admissions to a residential home, structural admissions to a nursing home, temporary admissions to a residential or nursing home, daytime treatment, day care, home care, mental healthcare service, social work, paramedical care and regular consultations with and visits from the general practitioner during a 12-month follow-up period.

### Data collection

Data for the effect evaluation in patients will be assembled by face-to-face interviews based on a questionnaire (including all validated measurement instruments) and in the formal caregivers by self-administered questionnaires. Trained interviewers, who are blinded for group allocation, will conduct the interviews and self-administered questionnaires at baseline, after six months and after 12 months.

Data for the process evaluation from patients, informal caregivers and health professionals are assembled by self-administered questionnaires and registration forms. To evaluate the patients’ and caregivers’ opinions about the care they received, a research assistant will conduct a semi-structured interview with all patients and informal caregivers separately to evaluate the care they received and to describe their experience of the care received in the rehabilitation programme. Furthermore, to evaluate the professionals’ opinions, a randomly selected representative sample of health care professionals will receive a questionnaire, which asks them about the programme being conducted in line with protocol, the possible reasons for deviations from protocol, the time invested, the bottlenecks identified and recommendations for improvement.

Furthermore, at the end of the intervention a focus group consisting of representatives of elderly stroke patients, informal caregivers, healthcare professionals and healthcare financiers will be organised to gather data about the implementation fidelity, programme deliverance and the opinions of the stroke professionals, patients and informal caregivers. Within the focus group semi-structured interview techniques will be used to discuss the questions about the rehabilitation programme as well as additional points raised by the participants. In order to check for contamination a selected sample of the electronic patient records will be analysed.

Data for the economic evaluation will be gathered by means of cost diaries, which are registered after six and 12 months. Healthcare costs are estimated according to the Dutch guideline for costs analyses in healthcare research [41].

### Sample size calculation

Using data from earlier research based on the Frenchay Activity Index score as primary outcome variable [42], an assumed clinically relevant difference in activity level of two stroke populations is at least 3.5 with a standard deviation of 8.9. Based on a power of 0.8 and an alpha of 0.05, the study would need a sample size of 102 patients in each group. With a drop-out to follow-up estimated at approximately 25%, each group should include 128 participants. In total 256 participants are needed for the study.

### Data analysis

The background characteristics of the participants will be described by using descriptive statistics. Baseline characteristics of the intervention and control group will be compared to detect differences at the start of the trial. Primary analyses of the effect data will be performed according to the intention-to-treat principle, including all participants with valid data on costs and clinical outcomes, regardless of whether they received the (complete) programme. Multiple regression analysis will be performed to calculate differences in the intervention and control group with regard to primary and secondary outcome measures. A per protocol subgroup analysis will be performed.

Data from the economic evaluation will be analysed to calculate cost-effectiveness and cost-utility ratios. Healthcare costs will be analysed by calculating incremental cost-effectiveness and cost-utility ratios. Data conducted from the process evaluation will be analysed by means of descriptive statistics and qualitative coding techniques. SPSS statistical software will be used for all analyses.

### Progress of the study

Implementation of the study protocol and the inclusion of participants started in October 2010 and will continue until September 2012. Data will be collected until September 2013. The first results of the study will be available in 2014.

## Discussion

This paper presents the study design of a multicentre randomised controlled trial to evaluate the effects and feasibility of a patient-tailored multidisciplinary rehabilitation programme for elderly stroke patients. The programme aims to improve care for elderly stroke patients who are admitted to a nursing home for neurorehabilitation. This study will provide information about the effectiveness, process and costs of the new multidisciplinary rehabilitation programme and will give insight into how the care of elderly stroke patients might be improved. If this trial shows effectiveness and cost-effectiveness of the rehabilitation programme, the aim is to implement the intervention into the Dutch health care system.

Some methodological and practical limitations concerning the current study exist. However, the presented design is the most feasible method to conduct data to evaluate the effectiveness of the intervention.

## Competing interests

The authors declare that they have no competing interests.

## Authors’ contributions

All authors participated in the design of the study. TPV is the main researcher and responsible for writing the study protocol. TPV, JCH, JAV and JMS made the plan for the study and developed the intervention. TPV, JCH, JAV, EJK and JMS all contributed to the organisation of the trial. JCH is the project leader of the study. JCH and JAV are the co-supervisors of TPV. JMS is the supervisor of TPV. TPV, JCH, JAV, EJK and JMS contributed to the writing of the study protocol. All authors read and approved the final version of the manuscript.

## Pre-publication history

The pre-publication history for this paper can be accessed here:

http://www.biomedcentral.com/1471-2377/12/164/prepub
